# The role of upstream sequences in selecting the reading frame on tmRNA

**DOI:** 10.1186/1741-7007-6-29

**Published:** 2008-06-30

**Authors:** Mickey R Miller, David W Healey, Stephen G Robison, Jonathan D Dewey, Allen R Buskirk

**Affiliations:** 1 Department of Chemistry and Biochemistry, Brigham Young University, Provo, UT 84602, USA

## Abstract

**Background:**

tmRNA acts first as a tRNA and then as an mRNA to rescue stalled ribosomes in eubacteria. Two unanswered questions about tmRNA function remain: how does tmRNA, lacking an anticodon, bypass the decoding machinery and enter the ribosome? Secondly, how does the ribosome choose the proper codon to resume translation on tmRNA? According to the -1 triplet hypothesis, the answer to both questions lies in the unique properties of the three nucleotides upstream of the first tmRNA codon. These nucleotides assume an A-form conformation that mimics the codon-anticodon interaction, leading to recognition by the decoding center and choice of the reading frame. The -1 triplet hypothesis is important because it is the most credible model in which direct binding and recognition by the ribosome sets the reading frame on tmRNA.

**Results:**

Conformational analysis predicts that 18 triplets cannot form the correct structure to function as the -1 triplet of tmRNA. We tested the tmRNA activity of all possible -1 triplet mutants using a genetic assay in *Escherichia coli*. While many mutants displayed reduced activity, our findings do not match the predictions of this model. Additional mutagenesis identified sequences further upstream that are required for tmRNA function. An immunoblot assay for translation of the tmRNA tag revealed that certain mutations in U85, A86, and the -1 triplet sequence result in improper selection of the first codon and translation in the wrong frame (-1 or +1) *in vivo*.

**Conclusion:**

Our findings disprove the -1 triplet hypothesis. The -1 triplet is not required for accommodation of tmRNA into the ribosome, although it plays a minor role in frame selection. Our results strongly disfavor direct ribosomal recognition of the upstream sequence, instead supporting a model in which the binding of a separate ligand to A86 is primarily responsible for frame selection.

## Background

Eubacteria possess a quality control system that rescues ribosomes stalled on defective mRNA templates. The key player in this system is transfer-messenger RNA (tmRNA), so called because it functions both as tRNA and mRNA. Aminoacylated tmRNA enters the empty A-site of stalled ribosomes and transfers alanine to the nascent polypeptide as would a normal tRNA. The ribosome then resumes translation with tmRNA as the template, directing the addition of another 10 amino acids to the nascent polypeptide [1]. As a result of tmRNA action, stalled ribosomes are released and recycled and the aborted protein product is tagged for destruction by proteases (for reviews, see [2-5]).

In the *trans*-translation model of tmRNA function described above, two steps occur that are difficult to reconcile with our current understanding of the mechanism of translation. The first is tmRNA entry into stalled ribosomes and its accommodation in the A-site despite the absence of a codon-anticodon interaction. In normal decoding, the ribosome monitors the geometry of the double helix formed by the codon and anticodon pair. Adenosines at positions 1492 and 1493 in the 16S ribosomal RNA form A-minor motif interactions with the A-form helix [6,7]. When the pairing is recognized as correct, elongation factor Tu hydrolyzes GTP, allowing the tRNA to be accommodated into the A-site and the chemistry of peptidyl transfer to proceed. tmRNA cannot participate in a codon-anticodon interaction; although it has a tRNA-like domain (TLD), it lacks an anticodon loop [8,9]. Furthermore, tmRNA can only enter ribosomal A-sites devoid of mRNA [10].

**Figure 1:**
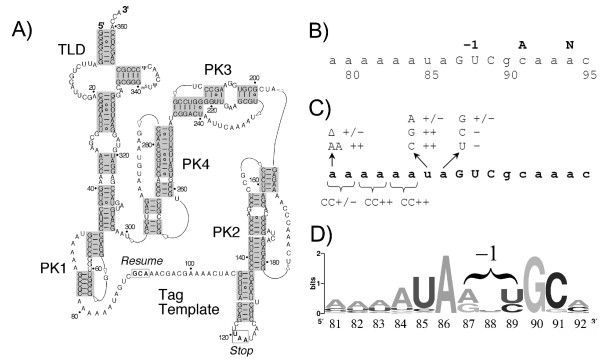
**Secondary structure of tmRNA and detail of the sequence upstream of the first codon of the tmRNA template**. (A) Secondary structure of *Escherichia coli *tmRNA, including the tRNA-like domain (TLD), four pseudoknots (PK1–4), with boxes surrounding the resume and stop codons at either end of the tag template [40]. (B) Fragment of a tmRNA sequence containing the -1 triplet (GUC) and resume codon (gca). (C) Results of the KanR assay [11] for independent mutants in the sequence upstream of the resume codon (see the text). ++ denotes survival at high stringency (30 μg/ml kanamycin at 37°C); +/- denotes survival at low stringency only (15 μg/ml kanamycin at 25°C); - denotes no survival at either stringency. (D) Sequence logo displaying the consensus of all 555 natural tmRNA sequences [21,41]. Created by Weblogo [42].

The second puzzling step in *trans*-translation is that the ribosome switches templates in the course of synthesizing a single polypeptide. How does the ribosome leave the stalled mRNA and resume elongation on tmRNA in the correct frame? Nearby structural elements contribute surprisingly little to positioning the first tmRNA codon in the decoding center. This 'resume' codon lies 12 nucleotides downstream from pseudoknot 1 (Figure 1A), which like the other three pseudoknots can be replaced by unrelated sequences without loss of activity [11]. Likewise, the tag template after the resume codon can be altered with little or no effect [12]. It appears that the crucial sequence acts locally: a five-nucleotide sequence immediately upstream of the resume codon sets the precise frame [13-15]. Mutation of this upstream sequence (UAGUC) leads to shifting of the frame of the tag sequence *in vitro *[13]. How the upstream sequence sets the frame on tmRNA is unknown. It may involve direct binding to the ribosomal decoding center or an interaction through another ligand.

A recent hypothesis put forward by Lim and Garber addresses both of these questions by proposing a function for the three nucleotides immediately preceding the resume codon [16]. They propose that this '-1 triplet' forms an A-form structure even in its unpaired state; this structure mimics the codon-anticodon interaction and binds directly to the ribosomal decoding center. In addition to allowing ribosomal entry and accommodation, this recognition event also establishes the reading frame on tmRNA. Following peptidyl transfer and translocation by one codon, the resume codon is moved into the A-site to act as a template for the continuation of translation.

Lim and Garber predict that not all 64 nucleotide triplets can assume an A-form conformation when single-stranded [16]. For 'forbidden' triplet sequences, the A-form conformation is dramatically destabilized because potential hydrogen bonds are not made. In earlier work, Lim and Curran showed that in the natural decoding process, mismatched codon-anticodon pairing leaves potential hydrogen bonding sites unsatisfied, preventing the formation of A-form helices [17]. In the case of the unpaired -1 triplet of tmRNA, conformational analysis predicts that large size differences (or steps) between adjacent bases prevent solvent molecules from satisfying all potential hydrogen bonds. Large steps are formed by sequences in which purines follow pyrimidines (for example, UG or CA). If A rotates to the *syn*-conformation, the size of the step is reduced: UA and CA are allowed unless followed by G (ruling out the triplet UAG).

According to these rules, the 18 forbidden triplets underlined in Table 1 cannot function as the -1 triplet in tmRNA [16]. Lim and Garber note that all natural tmRNA -1 triplet sequences are allowed by their rules. They also cite an earlier study in which 10 mutant -1 triplets were assayed *in vitro*, demonstrating that only the single forbidden triplet resumed some of the time in the wrong frame [13]. Although these facts fit their model, their hypothesis still lacks direct experimental support.

**Table 1:** Activity of tmRNA -1 triplet mutants in the KanR assay

UUU X	UCU X	UAU X	**UGU** X
UUC 10	UCC 100	UAC 25	**UGC** 100
UUA X	UCA 35	UAA X	**UGA** X
**UUG** X	**UCG** 100	**UAG** 100	**UGG** 50
CUU X	CCU X	CAU 40	**CGU** X
CUC 50	CCC 50	CAC 100	**CGC** 60
CUA X	CCA 100	CAA X	**CGA** X
**CUG** 100	**CCG** 100	**CAG** 90	**CGG** 20

AUU 90	ACU X	AAU 90	AGU 100
AUC 100	ACC 100	AAC 80	AGC 100
AUA 60	ACA 100	AAA 100	AGA 100
**AUG** 100	**ACG** 100	AAG 100	AGG 100

GUU 90	GCU X	GAU 100	GGU 100
GUC 100	GCC 100	GAC 100	GGC 100
GUA X	GCA X	GAA 95	GGA 100
**GUG** 100	**GCG** 25	GAG 100	GGG 100

tmRNA directs the addition of the last 15 amino acids of KanR onto ribosomes stalled on a *kanR *template in *Escherichia coli*. Only cells with functional tmRNA survive on 30 μg/ml kanamycin [11]. Each -1 triplet mutant was tested individually and the data reported as percentage survival on selective (kanamycin) media. Triplets listed as X showed no survival. Triplets forbidden by the rules developed by Lim and Garber [16] are underlined and in bold type.

The -1 triplet hypothesis is important because it is the most credible model for reading-frame selection that postulates direct binding of the ribosome to the tmRNA upstream sequence. Here we report a direct test of its predictions: we assayed all 64 possible sequences for tmRNA function. We show that the rules formulated by Lim and Garber do not accurately predict which -1 triplet mutants are functional in *Escherichia coli *cells. All of the mutants display measurable levels of tmRNA function *in vivo*, albeit at various levels. We conclude that the upstream sequence is not involved in the accommodation process, although the -1 triplet does play a minor role in setting the frame. These findings provide a counterargument to direct recognition of the upstream sequence by the ribosomal decoding center. Analysis of mistaken frame selection in several tmRNA mutants supports a model in which A86 is recognized by a ligand, placing the resume codon appropriately into the ribosomal A-site.

## Methods

### Plasmid construction

The *ssrA *gene in the plasmid p16Dum-Cat [11] was altered to express tmRNA mutants with every possible -1 triplet. The *ssrA *tag template sequence encodes the last 14 amino acids of the kanamycin resistance protein (KanR), ANKLQFHLMLDEFF, instead of the normal degradation tag, ANDENYALAA. p16Dum-Cat also expresses a truncated KanR protein lacking the C-terminal 15 amino acids, with the sequence Glu-Pro-Opal added to the C-terminus to induce ribosome stalling. Expression of this protein construct is driven from an arabinose-inducible promoter.

The 64 possible -1 triplet mutations were generated as a random library in the p16Dum-Cat vector. The oligonucleotide CAAGGTGCATGCCGAGGGGCGGTTGCCTCGTAAAAAGCCGCAAAAAATANNNGCAAATAAACTGCAGTTTCAT was synthesized with a random mixture of all four nucleotides at positions 87–89 of tmRNA (underlined). A short primer that anneals to the constant 3' region of this oligonucleotide was extended by the Klenow fragment of DNA polymerase I to generate double-stranded DNA. The resulting insert was digested with SphI and PstI, ligated into the p16Dum-Cat vector, and introduced into electrocompetent X90 ssrA::cat cells [1]. Clones from the resulting library were sequenced to identify plasmids expressing the 64 different -1 triplet mutant tmRNAs.

tmRNA expression vectors for the bacteriophage assays are derivatives of pKW11 [12]. Only the nucleotides listed are mutated in the tmRNA expressed from these plasmids; the rest of the sequence, including the tag template, is the same as wild-type *E. coli *tmRNA. The following mutations were incorporated separately into pKW11: UUG, UCG, UAG, UGG, or UGU as the -1 triplet, and the additional mutants U85A, and A86C. For example, in making the UUG mutant, primers 5pktrip: GCAAACGACGAAAACTACGCTTTAG and 3uug: CAATATTTTTTGCGGCTTTTTACGAGGCC were used to amplify the entire pKW11 plasmid in an inverse polymerase chain reaction (PCR). The blunt ends were phosphorylated with polynucleotide kinase and ligated together.

The plasmids that express tmRNA mutants for the frame misreading assays are based on pCH201, a derivative of pKW11 with a tmRNA tag template encoding ANDH_6_D [18]. The upstream sequence of pCH201 was altered to separately incorporate the following mutations: UGU or UUG as the -1 triplet, U85A, and A86C. Addition of the nucleotides GC before the tag sequence (G90) ensures that the His_6 _tag is added only if tmRNA is read by the ribosome in the -1 frame. For example, the oligonucleotides 5'his6: GCAAACGACCATCACCACCATCATCACGATTAATAAC and 3'gtc: GCGACTATTTTTTGCGGCTTTTTACGAGGCCAACC were used to amplify pCH201 in an inverse PCR, as above, to create the wild-type upstream sequence in the -1 frame plasmid. For the +1 frame plasmids, one extra nucleotide (G) was added before the resume codon such that the His_6 _tag is added only if tmRNA is read in the +1 frame.

### KanR assay for tmRNA activity

In the KanR assay, functional tmRNA molecules rescue ribosomes stalled on a truncated KanR protein and synthesize full-length KanR, rendering the cells resistant to kanamycin [11]. Bacteria containing each tmRNA mutant were grown overnight from a single colony in 2xYT with ampicillin. Cultures were diluted 10-fold into fresh media containing 2% arabinose and grown for 2 hours at 37°C to induce expression of the KanR protein. The cells were plated onto selective media: 2xYT, ampicillin, 2% arabinose, and 30 μg/ml kanamycin. Growth comparisons (selective versus non-selective plates) were made after incubation for 24 hours at 37°C. Select mutants were assayed again at lower stringency: 15 μg/ml kanamycin, 25°C and scored after 48 hours.

### Phage efficiency of plating assays

**Figure 2:**
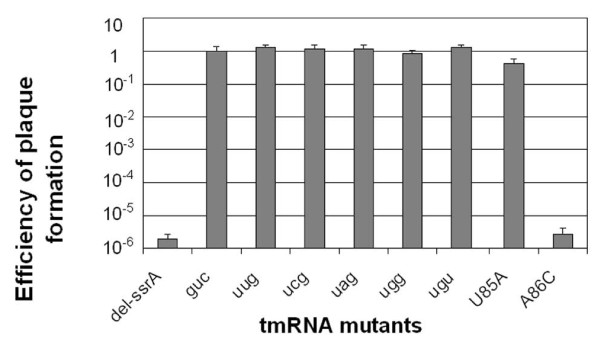
**Analysis of -1 triplet mutant activity**. The hybrid bacteriophage λ*immP22 c2-dis *only forms plaques on cells expressing active tmRNA [3,19]. Data are expressed as efficiency of plating (EOP) with wild-type tmRNA taken as EOP = 1. The del-ssrA mutant lacks tmRNA. Error bars represent the standard deviation of three independent experiments.

X90 ssrA::cat cells carrying -1 triplet mutants of tmRNA expressed from pKW11 were assayed for plaque formation using the phage λ*immP22 c2-dis *as described [11]. The number of plaques were counted for three different trials as reported in Figure 2.

### Western blot analysis of misreading

The -1 or +1 misreading vectors express tmRNA with frameshifted tags such that the ANDH_6_D tag is only added to stalled nascent peptides if the ribosome reads tmRNA in the correct frame. The plasmid pFLAG-MBP-SEP* expresses the complete maltose binding protein (MBP) containing an N-terminal FLAG tag and the amino acid sequence Glu-Pro-Opal added to the C-terminus, causing stalling during translational termination. X90 ssrA::cat cells containing one tmRNA plasmid and pFLAG-MBP-SEP* were grown in tetracycline and ampicillin to an OD_600 _of 0.5 and the expression of MBP induced with 1 mM IPTG. After 2.5 hours, the cells were pelleted and lysed with sodium dodecyl sulfate (SDS). Protein in the crude lysate was quantified via a Lowry assay and 12 μg of protein for each sample was resolved by 10% sodium dodecyl sulfate polyacrylamide gel electrophoresis (SDS-PAGE). The protein was transferred to polyvinylidenefluoride (PVDF) membrane and His_6_-tagged MBP was bound by a mouse anti-His_6 _antibody (Cell Signaling Technology). Binding of a rabbit anti-FLAG antibody (Sigma) to MBP was used to control for protein expression and loading (not shown). Fluorescent secondary antibodies (anti-mouse IRDye 800 and anti-rabbit IRDye 680, LI-COR Biosciences) were added and the blot was visualized with the Odyssey Infrared Imaging System (LI-COR Biosciences).

## Results

### In vivo KanR assay of all possible -1 triplet mutations in tmRNA

We examined the hypothesis that the tmRNA -1 triplet mimics a codon-anticodon helix and is recognized by the ribosome decoding center. According to the rules derived by Lim and Garber, 18 forbidden codons cannot assume the correct A-form structure as unpaired RNA (underlined in Table 1) [16]. We tested the function of tmRNA mutants corresponding to all possible -1 triplets. Our assay links tmRNA activity to the survival of *E. coli *cells by requiring *trans*-translation to complete the synthesis of a necessary protein. The tmRNA template sequence was altered to encode the last 14 amino acids of the kanamycin resistance protein (KanR). Ribosome stalling at the end of a truncated KanR protein leaves the cells sensitive to kanamycin unless tmRNA rescues the ribosome and directs the synthesis of full-length, active KanR protein. This assay was used previously to characterize mutants of tmRNA pseudoknot 1 and is a sensitive measure of *in vivo *tmRNA function [11].

*E. coli *cells containing a single -1 triplet mutant were plated onto selective media containing 30 μg/ml kanamycin at 37°C. Under these conditions, colonies with the wild-type *E. coli *-1 triplet sequence, GUC, survive equally well on selective versus non-selective plates (100%). Eighteen of the 64 mutant triplets showed no growth on selective plates, indicating decreased tmRNA activity (Table 1). Of these 18 dead mutants, only five had -1 triplet sequences that were forbidden by the rules described above. Nine other mutants showed weakened activity, from 10% to 50% survival. Of these nine, only three were forbidden. Conversely, eight of the triplets predicted to be forbidden displayed full activity (100%). We conclude from these data that the -1 triplet sequence does play a role in tmRNA activity, but that the rules derived by Lim and Garber do not accurately predict which sequences support tmRNA activity *in vivo*.

Several patterns in our data reveal the sequence determinants for -1 triplet function in tmRNA. The dominant pattern is that pyrimidines U and C are disfavored at nucleotide 87, the first of the -1 triplet. Of the 27 dead or weak mutants, 12 begin with U and 10 with C, but only 5 begin with a purine (A or G). At position 88 there seems to be little nucleotide bias in our data. At position 89, the last of the -1 triplet, U and A are disfavored (10 and 9 impaired mutants, respectively) compared with 4 each for C and G. The C89U mutation is particularly inactive when combined with U or C at position 87: of the eight YNU sequences, seven are dead and the other is weak. These two rules, the avoidance of pyrimidines at position 87 and U or A at position 89, explain the majority of the -1 triplet mutagenesis results.

In order to further characterize the activity of the 18 dead mutants, we repeated the assays at lower stringency: 15 μg/ml kanamycin at 25°C. Under these conditions, 15 of these 18 mutants showed 100% survival. CGA showed modest activity, but UGU and CGU remained completely inactive, with no colonies forming on the selective plates. These two mutants combine the ill effects of defying both patterns seen above, as they have a pyrimidine at position 87 and U at position 89. The fact that two -1 triplet mutants have no measurable activity in the KanR assay highlights the importance of these three nucleotides for tmRNA function. On the other hand, the fact that 61 of the 64 mutants show substantial tmRNA activity (at various levels) demonstrates that the role of this sequence is subtle, more likely fine-tuning function rather than the critical step of licensing tmRNA entry and accommodation.

### In vivo KanR assay of additional mutants in the upstream sequence

To identify other nucleotides that may play a role in accommodation or frame selection, we used the KanR assay to systematically test the role of the remaining nucleotides upstream of the resume codon. Between pseudoknot 1 and the coding sequence lies the 11 nucleotide sequence AAAAAAUAGUC, of which the final GUC sequence is the -1 triplet (Figure 1A and 1B). The surrounding pk1 structure [11] and coding sequence [12,13] can be altered without affecting the ability of tmRNA to rescue stalled ribosomes, suggesting that these 11 nucleotides contain the determinants for identifying the resume codon.

In accordance with their low conservation, the six adenosines at the 5'-end of this sequence tolerate alterations. To test the importance of distance constraints, we shortened (A84del) and lengthened (A84ins) the run of six A's by one nucleotide. Analysis in the KanR assay revealed that one extra A had no effect in the A84ins mutant. Deletion of one A resulted in weaker activity, with growth only at low stringency. (Hereafter mutants will be scored on their survival at low and high stringency; for example, +,- for the A84del mutant.) To examine sequence dependence, we mutated the A's in this sequence to C two nucleotides at a time. These mutants displayed modest (AA79CC +,-) or high activity (AA81CC and AA83CC both +,+); see Figure 1C. These results suggest that the sequence identity of the run of six A's is not critical and that changes in length are tolerated.

We mutated the highly conserved U85 and A86 to each of the three alternative nucleotides (Figure 1C). Mutation of U85 to C or G had no adverse affect (+,+) while the U85A mutation supported survival only on the low-stringency KanR assay (+,-). In contrast to the tolerance for changes at position 85, all mutations of A86 dramatically reduced activity. The A86G mutation survived only modestly (~50%) at low stringency (+,-). Mutation of A86 to pyrimidines C and U yielded no measurable activity in the KanR assay (-,-). Unlike single nucleotide mutations in the -1 triplet region, which had little or no effect, mutation of A86 alone destroys tmRNA activity. These results show that A86 plays a major role in tmRNA function, an observation consistent with the nearly complete conservation of A86 in tmRNA sequences (Figure 1D).

### Analysis of upstream mutants by bacteriophage plaque formation

To test the function of these tmRNA mutants in a second *in vivo *assay, we introduced mutations into otherwise wild-type tmRNA (encoding the natural tag ANDENYALAA) and assayed their ability to support phage plaque formation. The hybrid bacteriophage λ*immP22 c2-dis *only forms plaques on bacteria with intact *trans*-translation systems. Cells expressing wild-type tmRNA support the growth of around 10^5 ^more plaques than cells expressing no tmRNA [3,19]. In order to support phage growth, tmRNA mutants must be able to enter the ribosome, resume translation on the tmRNA template, and bring about termination at a stop codon [20]. The phage assay requires only release of the ribosomes, not proper frame choice or destruction of the aborted polypeptide [19].

Several -1 triplets that are forbidden by the rules of Lim and Garber were assayed for activity in the phage assay. The four UNG triplets were chosen because they exhibited varied levels of activity in the high-stringency KanR assay: UCG and UAG show 100% survival, while UGG was weaker at 50% and UUG showed no survival. All four of these mutant -1 triplets supported phage plaque formation as well as the wild-type control (Figure 2). We tested our most deleterious mutant, UGU, which did not survive in the KanR assay even under low-stringency conditions, and found that it too supported plaque formation as well as wild-type tmRNA. The wild-type level activity of these five forbidden triplets adds further evidence that the rules of Lim and Garber do not predict tmRNA activity *in vivo*. We conclude that the -1 triplet sequence is not required for any essential function of tmRNA in the phage assay, including tmRNA entry into the ribosome and accommodation into the A-site.

It appears that the phage assay is less sensitive than the KanR assay, in which subtle changes in levels of tmRNA activity were distinguished. This may be because tmRNA activity is close to the threshold required for survival in the KanR assay. In addition, small changes in tmRNA function caused by mutations are further amplified by the KanR enzymatic activity. In contrast, the threshold tmRNA function required for plaque formation in the phage assay is fairly low [20]. Another difference is that since the function of the tag peptide is not critical, proper frame selection is not required for phage propagation as, unlike in the KanR assay where it is required.

### Mutations upstream of the resume codon alter frame recognition on the tmRNA template

*In vitro *experiments show that mutations in the UAGUC sequence immediately upstream of the resume codon affect frame recognition. The U85A mutation, for example, causes the tag sequence to be read in the -1 frame 45% of the time *in vitro *[13]. To characterize frame misrecognition in living *E. coli *cells, we altered the tmRNA template sequence to encode ANDH_6_D, allowing tagging to be detected by an anti-His_6 _antibody. Two constructs were created: the first has two nucleotides inserted between the UAGUC sequence and the resume codon (GCA), such that the His_6_-containing peptide tag is only synthesized when tmRNA is read in the -1 frame. A second construct with a one-nucleotide insertion creates a template that produces the ANDH_6_D tag when read in the +1 frame. These tmRNA constructs allow visualization of tagging in the -1 or +1 frame for any given tmRNA mutant.

**Figure 3:**
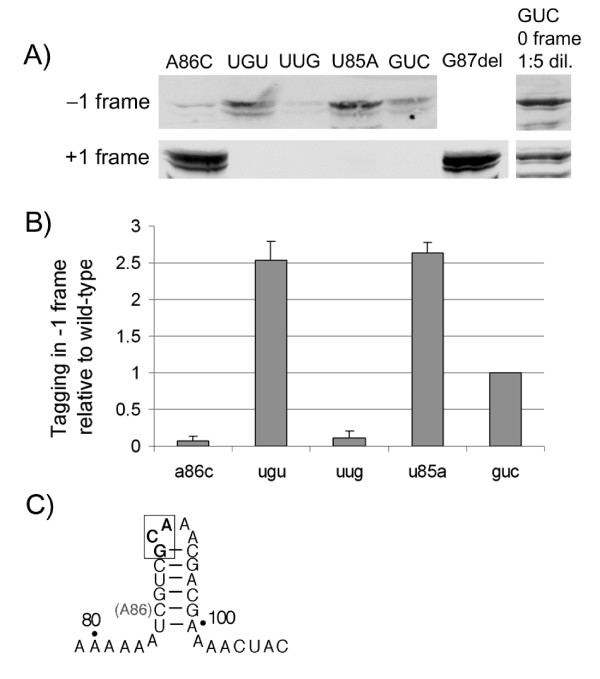
**Assay for misreading of the resume codon by tmRNA triplet mutants**. (A) tmRNA mutants were altered to encode the ANDH_6_D peptide tag provided that the ribosome resumes translation in the -1 (top) or +1 (bottom) frames. The maltose-binding protein with a stalling signal at the C-terminus served as a substrate for tagging, which was detected by anti-His_6 _antibodies. Addition of a known standard (tmRNA tagging in the 0 frame) at a 1:5 dilution allows comparison with the natural, 0 frame tagging process. (B) Quantification of tagging in the -1 frame relative to GUC (wild-type). Error bars represent the standard deviation of three independent experiments. (C) Proposed helical structure surrounding the resume codon (boxed) in the A86C mutant.

Three -1 triplet mutants of varying activity were analyzed: GUC (wild-type and +,+), UUG (+,-), and UGU (-,-), as well as the U85A (+,-) and A86C (-,-) mutations. The complete MBP with a stall-inducing sequence (Glu-Pro-Opal) at the C-terminus served as a substrate for tagging. The addition of the ANDH_6_D tag was monitored by immunoblot with anti-His_6 _antibodies. Analysis of tagging in the -1 frame vectors reveals one reason behind the low activities of the U85A and UGU mutants. As seen in previous *in vitro *studies, U85A exhibited increased translation in the -1 frame. Our results show both the U85A and UGU mutations lead to tag translation in the -1 frame at a 2.5-fold higher level than wild-type (Figure 3B). Comparison with a standard amount of tagged protein allows us to estimate that these mutants tag in the -1 frame at roughly 20% of wild-type tagging levels (Figure 3A).

Analysis of tagging in the +1 frame vectors shows no misreading, with one exception. The A86C mutant resumes translation in the +1 frame at a very high level, comparable to the G87 deletion mutant used as a positive control. Deletion of G87 restores the template sequence to the proper frame, negating the extra nucleotide added before the resume codon in the +1 frame vectors. Comparison with a known amount of tagged protein suggests that these mutants yield +1 tagged proteins about 50% as efficiently as wild-type tmRNA reading in the 0 frame (Figure 3A). This is a much stronger signal than seen for the -1 frame misreading above. This result is surprising because it was reported that the A86C mutant shows no activity whatsoever *in vitro *[13] and the A86C mutant showed no activity in the phage assay (Figure 2) as reported [15]. We speculate that these differences result from the different tag template sequences used for the various assays (discussed in the following).

## Discussion

The entry of tmRNA into stalled ribosomes to serve first as a tRNA and then as an mRNA template poses two problems for our current understanding of translation. First, how does tmRNA get past the decoding center when it lacks an anticodon and enters an A-site devoid of mRNA? Secondly, how does the ribosome resume translation on tmRNA at the proper site and in the proper frame? The -1 triplet hypothesis of Lim and Garber [16] suggests that three nucleotides immediately upstream of the resume codon mimic an codon-anticodon pair and bind directly to the ribosomal decoding center. Lim and Garber derived rules based on conformational analysis that predict which triplets can assume an A-form conformation as single-stranded RNA [16].

Using the KanR assay, which ties the life of *E. coli *cells to the function of tmRNA [11], we directly assayed each -1 triplet mutant for tmRNA activity. We found that eight of the 18 forbidden mutants showed activity indistinguishable from wild-type. Conversely, of the 18 mutants that were completely inactive, only five were predicted to be forbidden. Our data support the idea that the -1 triplet sequence is important to tmRNA function, but they do not fit the rules developed by Lim and Garber (Table 1). Instead, our data show that purines are highly favored at position 87 (the first of the -1 triplet) and that U and A are somewhat disfavored at position 89.

These characterizations broadly fit the natural distribution of -1 triplet sequences (Figure 1D). The preference for purines at position 87 matches the consensus obtained from all of the known natural tmRNA sequences [21], in which A and G occur roughly equally with only a negligible number of pyrimidines. There seems to be no nucleotide preference at the middle position in either our data or the consensus sequence. At position 89, C and U are equally represented in natural tmRNA sequences and strongly preferred to A or G. In our data, U is underrepresented at position 89 in active sequences. Perhaps this is because seven of the nine inactive sequences containing U89 have a pyrimidine at position 87. For example, the UGU and CGU mutants display no detectable activity even in the low-stringency KanR assay. Going against both of these trends appears to be particularly deleterious. As natural -1 triplets nearly always begin with purines, U89 may be more acceptable in the natural pool of sequences.

We performed a second assay based on the efficiency of plaque formation by the hybrid phage λ*immP22 c2-dis*. This phage can only form plaques on cells with active *trans*-translation systems [3,19]. We assayed five -1 triplet mutants ranging in the KanR assay from fully active to completely inactive under our lowest stringency conditions (UGU). We found that all five of these mutants had activities that were indistinguishable from wild-type tmRNA in the phage assay (Figure 2). All five of these -1 triplet sequences were forbidden by Lim and Garber's rules. The results of these two assays suggest that the -1 triplet sequence does not serve as a codon-anticodon mimic nor interact directly with the decoding center in a manner essential to tmRNA accommodation in the A-site.

A recent study by Shimizu and Ueda provides further evidence that the tmRNA -1 triplet sequence, and indeed the whole template sequence, is not required for ribosome binding, A-site accommodation, or peptidyl transfer [22]. Using a reconstituted translation system *in vitro*, they detected polyalanine synthesis with the SmpB protein and a tmRNA consisting of only the TLD. In this system, the growing polyalanine chain is passed from one tmRNA TLD to the next. The ability of tmRNA lacking a template sequence to bind ribosomes and transfer Ala proves that only the TLD and SmpB protein are required for the tRNA activity of tmRNA. In addition, the revised cryo-EM structure of SmpB and tmRNA bound to the 70S ribosome shows the SmpB C-terminus binding near the decoding center in the 16S rRNA [23]. Although the unstructured C-terminal tail is not visible in this structure, it is the most likely candidate for sending a signal equivalent to codon-anticodon pairing.

The -1 triplet is not required for legitimizing tmRNA entry into the ribosome, but together with U85 and A86, it does play a role in setting the correct frame for translation to resume. Analysis of tagging in the -1 frame reveals that both the deleterious UGU -1 triplet mutation and the U85A mutation increase translation in the -1 frame by a factor of 2.5 compared with wild-type (Figure 3B). The A86C mutation strongly induces +1 misreading. This tendency to select the wrong resume codon is one likely cause for the inactivity of these sequences in the KanR assay.

Analysis of the A86C mutation by phage assay (Figure 2) and *in vitro *tagging assays indicate that this mutation completely destroys tmRNA activity [13,15]. No ribosome release or tagging is seen in this mutant. These data contradict our finding that in the misreading assays the A86C mutant shifts the resume codon strongly to the +1 frame. The explanation lies in the fact that different tag template sequences were used in these assays. Chemical probing experiments show that the upstream region in the A86C mutant assumes a different structure, presumably a base-paired structure and therefore impervious to chemical probes [24]. A possible helical pairing surrounding the resume codon (between 85–90 and 95–100) would be further stabilized by the C:G pair created by the A86C mutation (Figure 3C). This structure could block access to a ligand that binds the upstream sequence. In our misreading assays, we used an altered tag sequence (ANDH_6_D) that cannot form this secondary structure.

Our findings confirm earlier work showing that A86 is the most important determinant for frame choice on the tmRNA template [14]. It is the most highly conserved nucleotide in the upstream sequence. U85 is tolerant to mutation, except for U85A. We propose that this mutation causes the A86 binding ligand to recognize A85 as well, shifting the frame back by one nucleotide. This explains the -1 shift in frame in U85A. The A86G mutation shows very low activity and the C and U mutations are totally inactive. When the masking effect of an inhibitory structure is removed, A86C causes +1 misreading because the A86 ligand binds to G87 instead, moving the resume codon ahead by one nucleotide. A86U has also been reported to induce +1 misreading *in vitro *[24]. The ligand for A86 prefers purines over pyrimidines. The -1 triplet also affects the frame but in a more subtle manner. The preference for a purine over a pyrimidine at the first base of this triplet (G87), the strongest trend for these three nucleotides, likely involves an additional interaction with the A86 ligand.

In contrast with an earlier model that suggested that the resume codon is positioned inside the ribosome by the global fold of tmRNA, and the frame fine-tuned by the upstream sequence [14], we propose that the A86-binding ligand acts independently to set the frame. The only ribosome binding element on tmRNA that can provide such positioning is the TLD. The four pseudoknots in tmRNA can be replaced with unrelated sequences and structures as can the tag template [11,25]. The distance from pseudoknot 1 to U85 is not critical for tagging, but insertions or deletions between A86 and the resume codon (G90) cause misreading of the resume codon [13]. It would seem that the A86-binding ligand is a ruler that establishes the frame four nucleotides down from A86 by placing the resume codon in the A-site to act as a template.

The refutation of the -1 triplet hypothesis discredits the most plausible model for direct binding of the ribosomal decoding center to the upstream sequence of tmRNA. The frame misrecognition results are best explained by a separate ligand binding to A86 and playing the dominant role in establishing the frame. What is the A86-binding ligand? One candidate is ribosomal protein S1, which was shown to crosslink to U85 in the upstream sequence (as well as pseudoknots 2 and 3) [26]. Visualization of tmRNA inside 70S ribosomes by cryo-electron microscopy revealed changes in the structure of the template sequence in ribosomes with or without the S1 protein [27,28]. Specifically, the template sequence is structured and removed from the decoding center in ribosomes lacking S1. The authors speculate that free S1 binds tmRNA and stabilizes a functional, open conformation of the template that is then passed to stalled ribosomes [27]. In support of this model, one study shows that S1 is dispensable for tmRNA entry and Ala transfer but required for its mRNA activity *in vitro *[29]. On the other hand, the expression of dominant negative S1 mutants *in vivo *does not interfere with tmRNA function although it inhibits normal translation [30]. *Bacillus subtilis *and other Gram-positive bacteria lack an S1 protein and yet have tmRNA and functional *trans*-translation systems. Two recent *in vitro *studies using *E. coli *or *Thermus thermophilus *reconstituted translations systems show that S1 does not affect tmRNA function [31,32]. S1 cannot interact directly with tmRNA on the ribosome, as S1 binds to the back of the head of the 30S subunit.

A second candidate is SmpB, a protein that enhances aminoacylation of tmRNA and is required for entry into the ribosome (as discussed above). The best characterized binding site of SmpB is on the TLD of tmRNA, the interaction visualized by two co-crystal structures of SmpB and short fragments of tmRNA [33,34]. Multiple copies of SmpB can bind tmRNA simultaneously [35,36], however, even in the context of the 70S ribosome [23]. SmpB binding also alters the accessibility of the upstream sequence and pseudoknot 1 to nucleases in probing experiments, leading to the proposal that it plays a role in frame choice [36]. This possibility is further strengthened by the recent finding that SmpB binding protects U85 from chemical modification and that this protection shifts by one nucleotide in tmRNA mutants that induce misreading of the resume codon [24]. On the other hand, several crosslinking, chemical probing, and hydroxyl-radical cleavage assays have failed to detect such an interaction [35,37-39]. It is not known which amino acids on SmpB may interact with the upstream sequence. Through this interaction, SmpB may orient the template sequence with respect to the TLD, causing conformational changes in the template, upstream sequence, and pseudoknot 1, positioning the resume codon in the A-site. If this model is correct, SmpB is solely responsible for allowing tmRNA to enter stalled ribosomes, tricking the decoding center, and it also plays a crucial role in its interaction with the upstream sequence on tmRNA to set the frame for translation of the tmRNA template.

## Conclusion

Our findings disprove the -1 triplet hypothesis and strongly disfavor a model in which ribosome binding selects the reading frame in tmRNA directly. Binding of a separate ligand, perhaps S1 or SmpB, to A86 is primarily responsible for frame choice, although other upstream sequences including U85 and G87 play a minor role. Mutations in these nucleotides lead to translation of the tmRNA in alternate frames *in vivo*.

## Authors' contributions

MRM carried out the KanR assay and phage experiments for the -1 triplet mutants. DWH performed the Western blots to reveal tagging in different frames and developed the model to explain the results. SGR designed and tested the upstream mutants in the KanR assay and together with JDD developed the frame misrecognition immunoblot assay. ARB designed and directed the study and wrote the manuscript. All authors read and approved the final manuscript.
